# A Simple, Centrifugation-Free, Sperm-Sorting Device Eliminates the Risks of Centrifugation in the Swim-Up Method While Maintaining Functional Competence and DNA Integrity of Selected Spermatozoa

**DOI:** 10.1007/s43032-020-00269-5

**Published:** 2020-07-30

**Authors:** Huidrom Yaiphaba Meitei, Shubhashree Uppangala, Krishna Sharan, Srinidhi Gururajarao Chandraguthi, Arunkumar Radhakrishnan, Guruprasad Kalthur, Stefan Schlatt, Satish Kumar Adiga

**Affiliations:** 1 Department of Clinical Embryology, Kasturba Medical College, Manipal, Manipal Academy of Higher Education, Manipal, 576104 India; 2 Department of Radiation Oncology, Kasturba Medical College, Manipal, Manipal Academy of Higher Education, Manipal, 576104 India; 3 SAR Healthline Pvt. Ltd., Chennai, 600010 India; 4 Centre of Reproductive Medicine and Andrology, Albert-Schweitzer Campus 11, 48149 Münster, Germany

**Keywords:** Sperm preparation, DNA damage, Migration–sedimentation, Sperm function, Swim-up, MIGLIS

## Abstract

This pilot study was conducted to explore the benefits of using a centrifugation-free device based on the migration–sedimentation (MS) technique over centrifugation-based techniques in selecting competent spermatozoa, as compared with using split human semen samples. Ejaculates from 35 men undergoing semen analysis were split into four parts where one part was retained as the neat (NE) and the other three parts were subjected to sperm selection by using migration–sedimentation (MS), density gradient (DG) separation, and swim-up (SU) techniques. Sperm functional characteristics along with mitochondrial integrity, tyrosine phosphorylation, acrosome reaction, and ultrastructure were measured. The ability of selection techniques in reducing spontaneous and radiation-induced sperm DNA lesions was assessed by the TUNEL assay. In results, MS-selected spermatozoa had higher viability (*P* < 0.001), longevity in terms of total motility at the end of 6 and 18 h post-extraction (*P* < 0.001), and mitochondrial integrity (*P* < 0.001) compared with those selected by DG. Furthermore, spontaneous DNA lesions were significantly reduced in MS and SU fractions compared with NE (*P* < 0.001). Similarly, radiation-induced sperm DNA lesions were significantly lower in MS and SU fractions (*P* < 0.001) compared with DG. Ultrastructural analysis using scanning electron microscopy suggested a moderate, non-significant increase in the number of spermatozoa with normal head and mid-piece in MS fraction compared with other methods. In conclusion, the MS-based device offers a centrifugation-free, efficient, and reliable sperm selection method, making it suitable for partially equipped intra-uterine insemination (IUI) laboratories or office IUI programmes. Further research should focus on the safety and clinical usefulness of the device in assisted conception programmes in general and IUI in specific.

**Electronic supplementary material:**

The online version of this article (10.1007/s43032-020-00269-5) contains supplementary material, which is available to authorized users.

## Introduction

Centrifugation-based sperm preparation techniques primarily involve centrifugal pelleting of spermatozoa in the liquified ejaculate followed by selection of healthy sperm. The swim-up technique (SU) is routinely used either singly or in combination with density gradient (DG) centrifugation for the selection of the most active and motile spermatozoa [1, 2]. Both methods provide clean population of spermatozoa; however, it does not replicate the complex selection processes seen in in vivo methods. [3] Importantly, centrifugation-based techniques are reported to result in sperm damage and subsequent iatrogenic failure of pregnancy in some patients [4]. Impairment in sperm motility [5], mitochondrial damage [6], and DNA damage [7, 8] are observed with centrifugation-based sperm preparation methods. Reactive oxygen species (ROS) generated during the mechanical force of centrifugation is suggested to impair the structural and functional integrity of sperm cells [9, 10].

In order to prevent centrifugation-induced damage on spermatozoa, attempts are made to develop centrifugation-free devices to extract spermatozoa [11–13]. Based on the motility and by the help of gravity, sperm cells can sediment in the bottom of a tube without centrifugation. Thus, migration–sedimentation (MS) of spermatozoa are expected to avoid deleterious effects of centrifugation. A recent study has shown improved motility in the sperm fraction extracted by using the MS technique over other conventional methods [14].

MS is an old technique; [15, 16] however, its benefits to apply in assisted fertilization is not established with adequate fundamental scientific evidence. Due to rising concerns about the iatrogenic damage to spermatozoa, here, we explored the benefits of the centrifugation-free MS technique using a simple commercially available device for use in assisted fertilization programmes. We sought to determine if the MS method improves the selection of functionally competent, morphologically normal, and DNA-intact spermatozoa over standard centrifugation-based techniques in split human semen samples. A commercial kit designed on the migration–sedimentation principle was used in the study along with other conventional separation techniques.

## Materials and Methods

### Study Subjects

This pilot study included patients (*N* = 35) undergoing semen analysis as a part of infertility workup at the university infertility clinic. Institutional Ethical Committee approval was obtained (IEC 689/2018) before the initiation of the study. The patients who agreed to participate and signed the informed consent were included. Depending on the semen characteristics [17], only normozoospermic ejaculates with a minimum of 80 million total sperm number were included. Semen characteristics of the study subjects are shown in Table 1. Post–semen analysis, left over samples were split into four parts where one part was retained as neat (NE) and other three parts were subjected to sperm selection using DG separation, MS, and SU. The recovery rate was calculated as described earlier [18].

**Table 1:** Age and semen profile of the study subjects

	Mean ± SEM (*N* = 35)
Patient age (in year)	36.4 ± 0.7
Semen volume (mL)	3.3 ± 0.2
Sperm concentration (10^6^/mL)	63.1 ± 4
Total sperm number (10^6^/ejaculate)	208.7 ± 20.6
Total motility (%)	67.5 ± 2.1
Progressive motility (%)	44.2 ± 1.8
Normal forms (%)	12.7 ± 2.2
Viability (%)	61.9 ± 2.1

Out of 35 samples, 15 samples were X-irradiated in the radiation facility to induce DNA fragmentation in spermatozoa in order to understand the efficiency of selection techniques in eliminating DNA-fragmented spermatozoa. Irradiation of liquefied ejaculate on a petri dish was performed immediately (within 10 min) using a linear accelerator (Versa HD, Elekta, 2013) with X-ray energy of 6 MV at a dose rate of 5 Gy per minute. A total of 10 Gy was delivered onto the ejaculate at room temperature.

### Migration–Sedimentation–Based Device

**Fig. 1:**
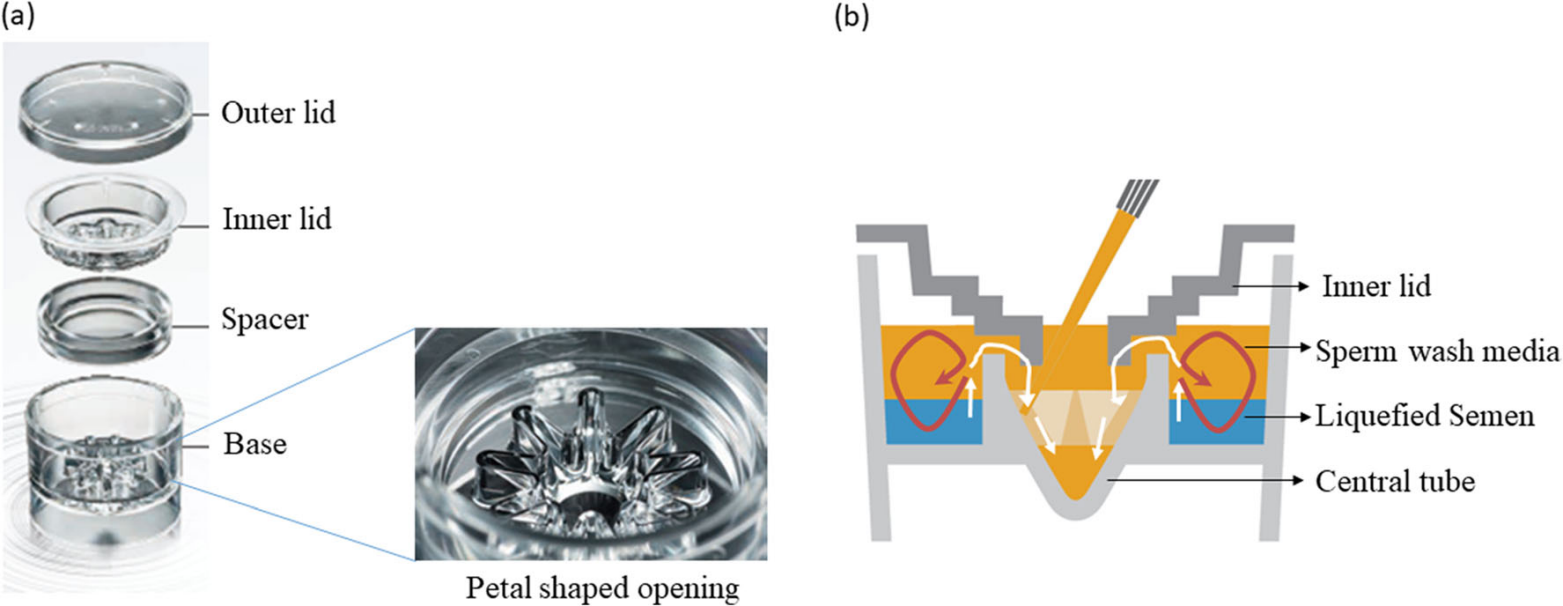
Migration–sedimentation device. **a** Photograph showing various parts of the device. **b** Schematic drawing of the device demonstrating the sedimentation of motile spermatozoa into the central tube indicated by the white arrow. Non-motile spermatozoa, debris, and round cells would stay in the outer well (red arrow)

The device used in this study works on the principle of MS that was originally developed by Tea et al. [19] The device is made up of transparent bio-compatible Cyclo olefin polymer developed by Menicon Life Science, Japan. The device has four parts comprising of an outer lid, inner lid, spacer, and base as shown in Fig. 1a. The base consists of a central tube inserted inside a tubular container, which has a petal-shaped opening. According to the manufacturer, the liquefied semen/diluted sample (maximum of 3 mL) is deposited into the outer well (sample injection space) of the base, and sperm wash medium is added into the central tube after placing the inner lid until it overflows and covers the ejaculate. During incubation, only motile spermatozoa will jump over the edge of the central tube, and the sediment was at the bottom of the central tube as shown in the Fig. 1b. The petal-shaped opening increases the surface area and helps in sedimentation of more motile spermatozoa. The spacer is helpful while using low-volume ejaculates. The inner lid prevents the mixing of immotile sperm and debris with motile spermatozoa due to convection.

### Sperm Selection by Using the MS Device

One part of the split fraction was diluted up to 1.5 mL using sperm wash media (Earle’s balanced salt solution, Cat. No. M5017, Sigma-Aldrich, USA) supplemented with sodium bicarbonate (Cat. No. S5761, Sigma-Aldrich, USA) and 0.1% bovine serum albumin (BSA) and dispensed into the outer well after placing the spacer inside the base. Then, the inner lid was placed onto the diluted semen. After ensuring that the inner lid was properly fixed into the grove of the base, 2 mL of pre-warmed sperm wash media was dispensed into the central tube of the base so that the semen was completely covered with sperm wash media. The outer lid was placed on the base, and the entire unit was incubated at 37 °C and 5% CO_2_. After 1 h, 0.4 mL of the sperm suspension was collected from the central tube to use for further analysis.

### Sperm Selection by Using DG

The split fraction was overlaid on the 90% and 45% gradient solution layers (Sil-Select Plus, Cat. No. SIP100, FertiPro N.V., Belgium) and then centrifuged at 500*g* for 20 min. The pellet was re-suspended in 1 mL of pre-warmed EBSS medium supplemented with 0.1% BSA and centrifuged at 300*g* for 8 min. Additional washing was performed at 200*g* for 8 min before the pellet was suspended in 0.4 mL of EBSS media supplemented with sodium bicarbonate and 0.1% BSA.

### Sperm Selection by Using SU

Split fraction was mixed with 1 mL of pre-warmed EBSS medium supplemented with 0.1% BSA and centrifuged at 300*g* for 8 min. Additional washing was performed at 200*g* for 8 min before the pellet was overlaid using 0.4 mL of EBSS media supplemented with sodium bicarbonate and 0.1% BSA. The tube was positioned at a 45° angle and incubated at 37 °C and 5% CO_2_. After 1 h, 0.4 mL of the sperm suspension was collected to use for further analysis.

### Mitochondrial Membrane Potential

Mitochondrial membrane potential was evaluated in spermatozoa as described earlier [20] with minor modifications. Approximately 0.5 million spermatozoa were incubated with 10 μg/mL of rhodamine 123 (Cat. No. R8004, Sigma-Aldrich, USA) at 37 °C and 5% CO_2_ for 20 min. After washing, a drop of sperm suspension was placed on a glass slide and observed under fluorescent microscope (Imager-A1; Zeiss, Gottingen, Germany). Spermatozoa exhibiting bright fluorescence only at the mid-piece region was considered to have intact mitochondria (Supplementary figure 1). A minimum of 500 spermatozoa were scored from each sample, and results are expressed as the percentage of spermatozoa with damaged mitochondria.

### Sperm Viability

Viability was determined by using the eosin–nigrosine staining method [17]. One drop of sperm sample was mixed with 2 drops each of 1% eosin Y and 10% nigrosin solutions; after thorough mixing, a smear was made on a pre-cleaned glass slide and air-dried. Viability was assessed using a compound microscope at × 100 magnification. A minimum of 200 spermatozoa were counted to determine the percentage of viable cells. Viable spermatozoa appear unstained, whereas non-viable cells appear pink in colour.

### Tyrosine Phosphorylation

Tyrosine phosphorylation in spermatozoa was determined by immunofluorescence as previously published [21] with minor modifications. Spermatozoa from each group were fixed in chilled methanol:acetone (1:1) for 10 min at − 20 °C followed by blocking using 1% BSA and air-dried. Cells were washed in phosphate buffered saline (PBS) and incubated with mouse monoclonal 4G10® Platinum, Anti-Phosphotyrosine Antibody, 1:200 dilution (Cat. No. 05-1050X, Sigma-Aldrich, USA), overnight at 4 °C in a moist chamber. Cells were washed in PBS followed by incubation with FITC-conjugated goat anti-mouse IgG secondary antibody 1:1000 dilution (Cat. No. NB7538, Novus Biologicals, USA) for 30 min at 4 °C. Counterstaining was done with 4′,6-diamidino-2-phenylindole (DAPI; 2 μg/mL; Cat. No. D9542, Sigma-Aldrich, USA). The spermatozoa were mounted using Dako mounting medium (Cat. No. S3023, DAKO) and observed under fluorescence microscope at × 100. A minimum of 200 cells were scored to calculate the percentage of spermatozoa displaying high-intensity fluorescence at the various regions of spermatozoa (acrosome cap, equatorial region, mid-piece, and tail) indicating the extent of sperm capacitation (Supplementary figure 2).

### Acrosome Reaction

The ability of spermatozoa to undergo acrosome reaction was assessed by previously described calcium ionophore (A23187)–induced acrosome reaction (CIAR) assay [22] with minor modifications. Sperm suspension was treated with or without 5 μM calcium ionophore (Cat. No. C7522, A23187, Sigma-Aldrich, USA) for 30 min at 37 °C and 5% CO_2_. Post-incubation, washed cells were smeared on the coverslip permeabilized in 100% cold methanol. Cells were stained with FITC-conjugated *Pisum sativum* agglutinin (FITC PSA; Cat. No. L0770, Sigma-Aldrich, USA) at a concentration of 25 μg/mL at room temperature for 30 min. Washed sperm cells were then counterstained with 7 μg/mL of propidium iodide (PI; P4170, Sigma-Aldrich, USA) and mounted on a clean slide using Dako mounting medium. A minimum of 500 spermatozoa were evaluated under a fluorescence microscope (Imager-A1; Zeiss, Gottingen, Germany). Acrosome-reacted spermatozoa had no green acrosome cap (Supplementary figure 3).

### Terminal Deoxynucleotidyl Transferase dUTP Nick End Labelling Assay

Terminal deoxynucleotidyl transferase dUTP nick end labelling (TUNEL) assay was performed as per our previously published protocol [23]. Concisely, sperm cells were fixed on a poly-l-lysine–coated slide using 4% paraformaldehyde for 1 h and then permeabilized by 0.1% Triton X-100 in 0.1% sodium citrate solution in PBS. After 1-h equilibration at room temperature, cells were incubated with TUNEL reaction mixture (Cat. No. 12156792910, Roche Diagnostics, USA) at 37 °C for 1 h in dark. The cells were washed and stained with 2 μg/mL DAPI (P4170, Sigma-Aldrich, USA) for 1 min and mounted on a clean slide. The cells were observed under a fluorescence microscope (Imager-A1; Zeiss, Gottingen, Germany) at × 40 magnification. The nucleus of the TUNEL-labelled spermatozoa displayed red fluorescence (Supplementary figure 4). A minimum of 1000 spermatozoa were scored, and the labelling index was determined.

### Shorr Staining

Sample containing approximately 0.1 million spermatozoa was smeared, air-dried, and stained as per the WHO protocol [17]. The stained slides were mounted using DPX and observed at × 100 magnification under oil immersion. A minimum of 100 cells were assessed, and the percentage of morphological variations were recorded.

### Scanning Electron Microscopy

Ultrastructural sperm morphology was analyzed using scanning electron microscopy (SEM) as described earlier [24] with minor modifications. Briefly, sperm cells were centrifuged and fixed on a clean slide using 2.5% glutaraldehyde in 0.1 M sodium acetate buffer (pH 7.3) for 1 h. The fixed cells were washed using 0.1 M sodium acetate and subsequently dehydrated using increasing concentration of ethanol (30, 50, 70, 80, 90, and 100% for 10 min each). Following dehydration, the cells were air-dried and subjected to critical point drying using carbon dioxide. Finally, the cells were sputtered using gold and observed under field emission SEM (FESEM; Carl Zeiss Sigma, Germany) at × 20,000 magnification. A minimum of 100 cells were analyzed for each data point as per the previously published criteria [24].

## Statistical Analysis

The statistical significance for the variables was calculated by using either repeated measures analysis of variance (ANOVA) for normal distribution or Friedman’s test as a nonparametric method in case failing the normality test. The statistical tests were done using the GraphPad InStat 3.0 statistical package (GraphPad Inc., USA). All the graphs were plotted using Origin 8.0 (Origin Lab Corporation, Northampton, MA, USA). *P* < 0.01 was considered statistically significant.

## Results

**Fig. 2:**
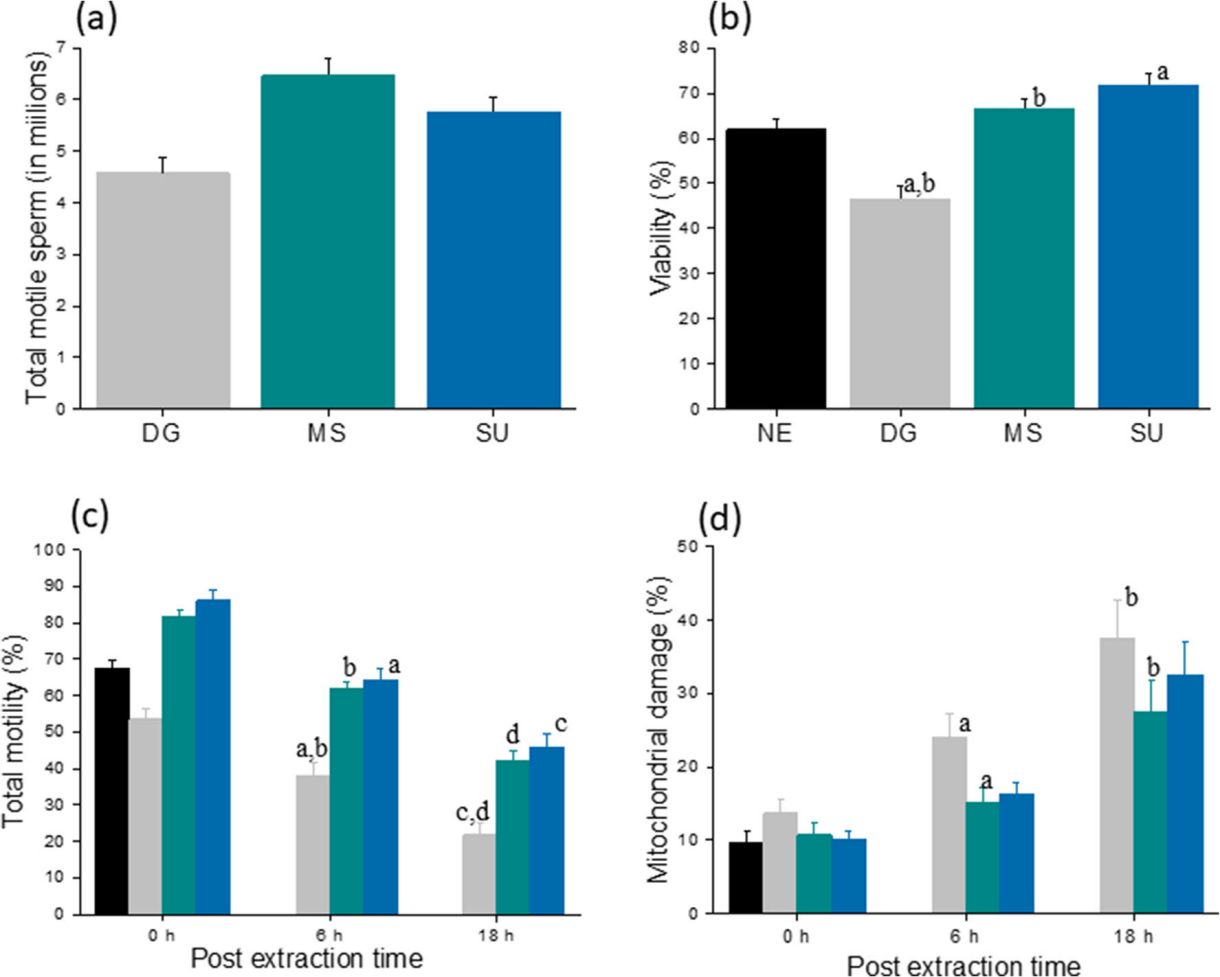
Sperm functional characteristics in the processed fraction. **a** Total motile sperm (TMS in millions). Please note that the values are not significantly different between the groups. **b** Sperm viability across various groups. *P* < 0.001 across identical letters (a, b). **c** Total motility immediately after selection and after 6 and 18 h. *P* < 0.001 across identical letters (a, b, c, d) in the same group. **d** Percent mitochondrial damage at various post-selection time periods. *P* < 0.001 between the groups with the letter ‘a, b’. The bar represents mean, and the error bar represents standard error of the mean (SEM)

Ejaculate fractions containing equal numbers of spermatozoa were subjected to three sperm selection techniques. The total motile sperm (TMS) was comparable between MS and SU, whereas DG had a marginally lower TMS (Fig. 2a). The values for TMS and recovery rate are shown in Table 2. The recovery rate was found to be maximum in MS (48.5 ± 2.7) compared with SU (43.6 ± 2.9) and DG (34.9 ± 3.1). However, the difference was significantly different only between DG and MS (*P* < 0.001). On the other hand, viability was significantly higher in MS- (66.4 ± 2.1) and SU- (71.6 ± 2.8) extracted spermatozoa compared with that of DG (46.6 ± 2.9; *P* < 0.001) (Fig. 2b). When the longevity (in terms of total motility) of the processed spermatozoa was assessed at the end of 6 h, spermatozoa extracted by both MS and SU retained approximately 65% motility, which was significantly higher than DG (37.9 ± 3.4; *P* < 0.001). A similar trend was observed at the end of 18 h where only about half of the spermatozoa processed by DG showed motility (21.6 ± 3.2) significantly lower than MS (42.2 ± 2.6) and SU (45.9 ± 3.4) groups (*P* < 0.001) (Fig. 2c). The mitochondrial function of the spermatozoa is important for sperm motility and longevity, and was assessed at different time points. Spermatozoa isolated by MS had significantly lower number of damaged mitochondria at the end of 6 and 18 h (15.06 ± 2.0 and 27.46 ± 4.4 respectively) compared with those by other groups; however, statistically, a significant difference was found only with DG at 6 and 18 h (24.13 ± 3.1 and 37.6 ± 5.1; *P* < 0.001) (Fig. 2d).

**Table 2:** Total motile sperm and recovery rate observed across three study groups

Parameter	Pre-wash	Sperm selection methods		
		DG	MS	SU
Motility (%)	67.3 ± 2.1	53.4 ± 2.9^a,b^	81.6 ± 1.6	86 ± 2.9
TMS (× 10^6^ sperm)	13.4 ± 0.4	4.5 ± 0.2	6.4 ± 0.3	5.7 ± 0.2
Recovery rate (%)	NA	34.9 ± 3.1^a^	48.5 ± 2.7	43.6 ± 2.9

*TMS*, total motile sperm; *DG*, density gradient; *MS*, migration–sedimentation; *SU*, swim-up; *NA*, not applicable

All values are presented as mean ± SEM

^a^*P* < 0.001 vs MS

^b^*P* < 0.001 vs SU

**Fig. 3:**
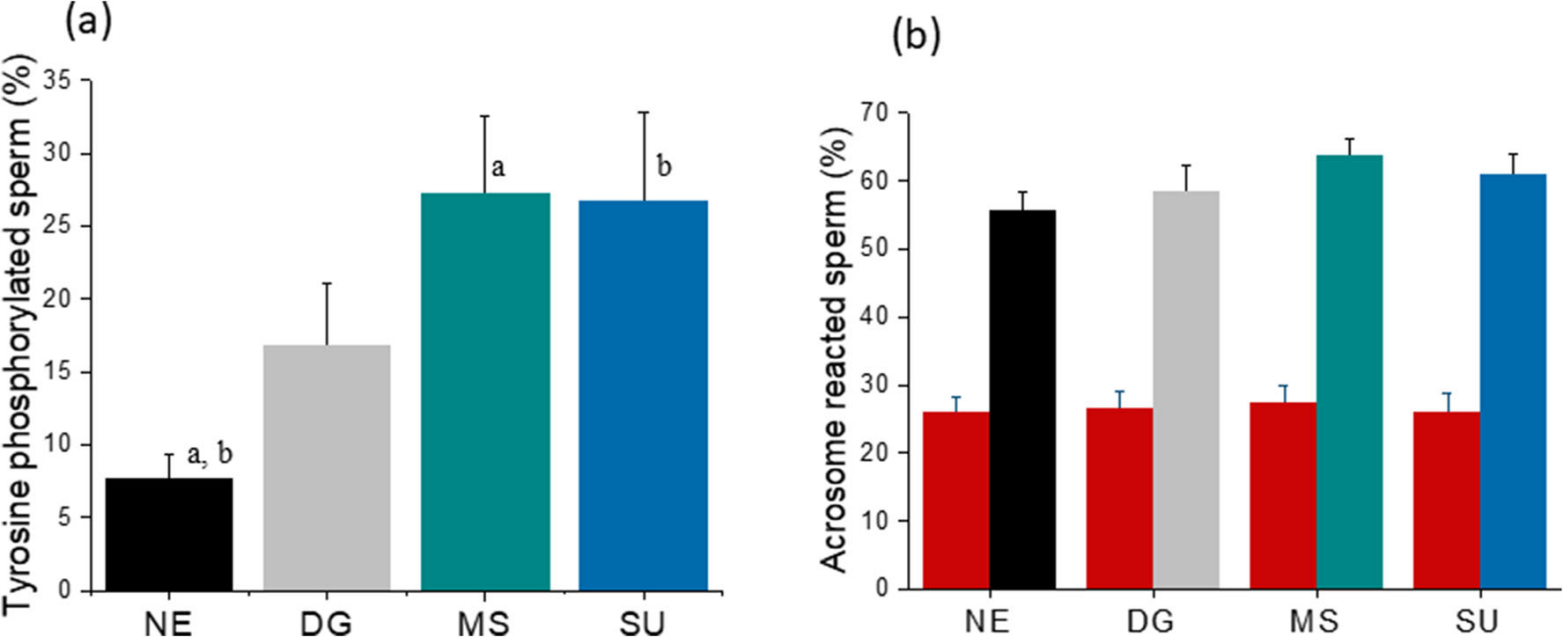
**a** Immunostaining of spermatozoa displaying tyrosine phosphorylation. *P* < 0.001 between the groups with the identical letters (a, b). **b** Acrosome-reacted spermatozoa. Red bar: spontaneously reached in corresponding groups; grey bar: DG; teal bar: MS; blue bar: SU calcium ionophore–induced acrosome reaction (CIAR) in spermatozoa. Please note that the values are not significantly different between the groups. The bar and error bar represent mean and the standard error of the mean (SEM) respectively

The functional competence of extracted spermatozoa was assessed for capacitation and acrosome reaction. Number of spermatozoa displaying protein tyrosine phosphorylation, an indicator for capacitation, was significantly higher in MS (27.2 ± 5.3) and SU (26.7 ± 6.1) fractions compared with NE (*P* < 0.001), whereas DG (16.8 ± 4.3) had lower number of positive sperm compared with MS and SU; however, the differences were not significant (Fig. 3a). On the other hand, CIAR assay demonstrated comparable number of acrosome-reacted spermatozoa across all three groups (Fig. 3b).

**Fig. 4:**
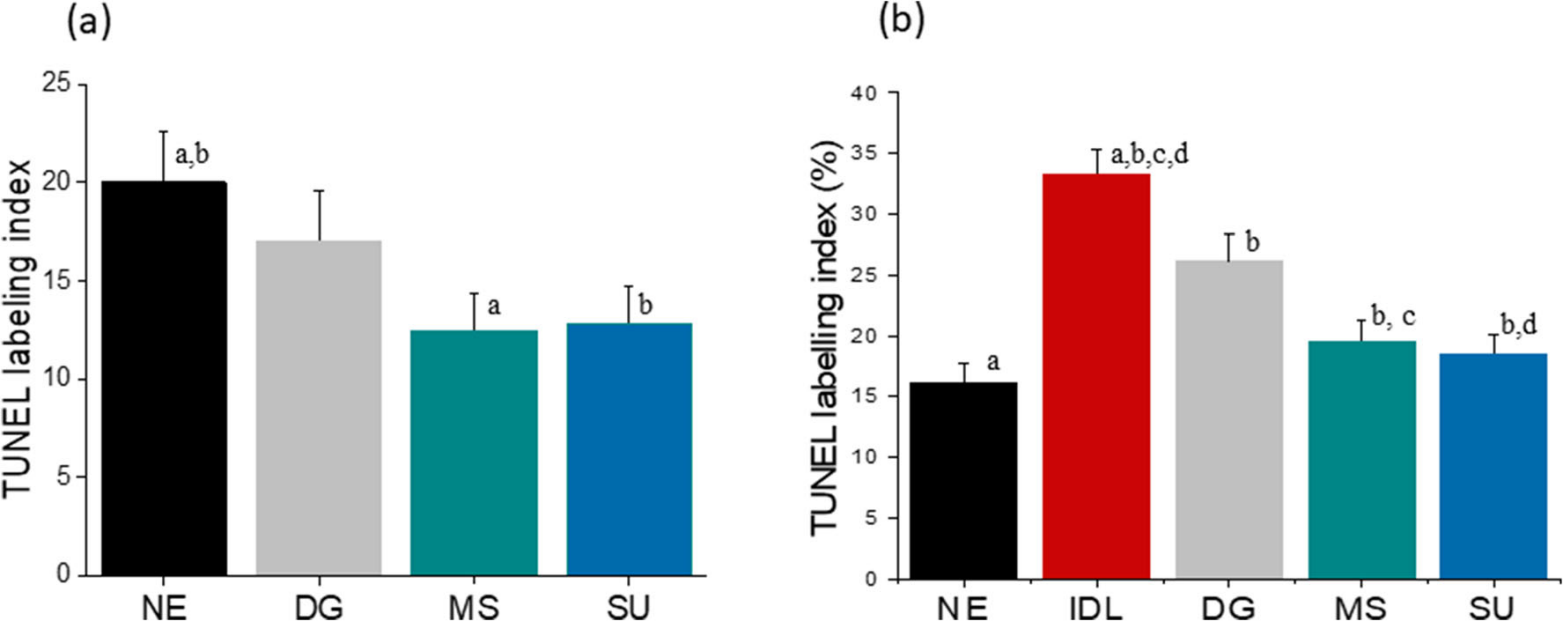
Sperm DNA fragmentation in the processed fraction. **a** The ability of various techniques in eliminating spontaneous DNA fragmentation from the ejaculate. Black bar: NE; grey bar: DG; teal bar: MS; blue bar: SU. *P* < 0.001 between the groups with the identical letters (a, b). **b** The ability of various techniques in eliminating radiation-induced DNA fragmentation from the ejaculate. *P* < 0.001 between the groups with the identical letters (a, b, c, d). Data were presented as mean ± SEM

In order to test the ability of MS in extracting DNA-intact spermatozoa, two different approaches were attempted. Initially, spermatozoa from all three methods were evaluated for TUNEL and compared with that of NE. The TUNEL index in spermatozoa post-MS and -SU was reduced to half in relation to NE (*P* < 0.001). Interestingly, a moderate but non-significant reduction in labelling index was observed in DG-processed sperm fraction in comparison with NE (Fig. 4a). Alternately, spermatozoa carrying radiation-induced DNA lesions (IDL) with approximately double the number of TUNEL-positive cells (33.3 ± 1.9; *P* < 0.001 vs NE) were subjected to DG, MS, and SU methods to understand the efficiency of these techniques when DNA damage level is high in the ejaculates. The labelling index in DG, MS, and SU reduced significantly post-selection (26.1 ± 2.2, 19.5 ± 1.7, and 18.5 ± 1.5 respectively; *P* < 0.001), whereas labelling index in MS and SU further demonstrated a reduction compared with DG-extracted spermatozoa (*P* < 0.001) (Fig. 4b).

**Fig. 5:**
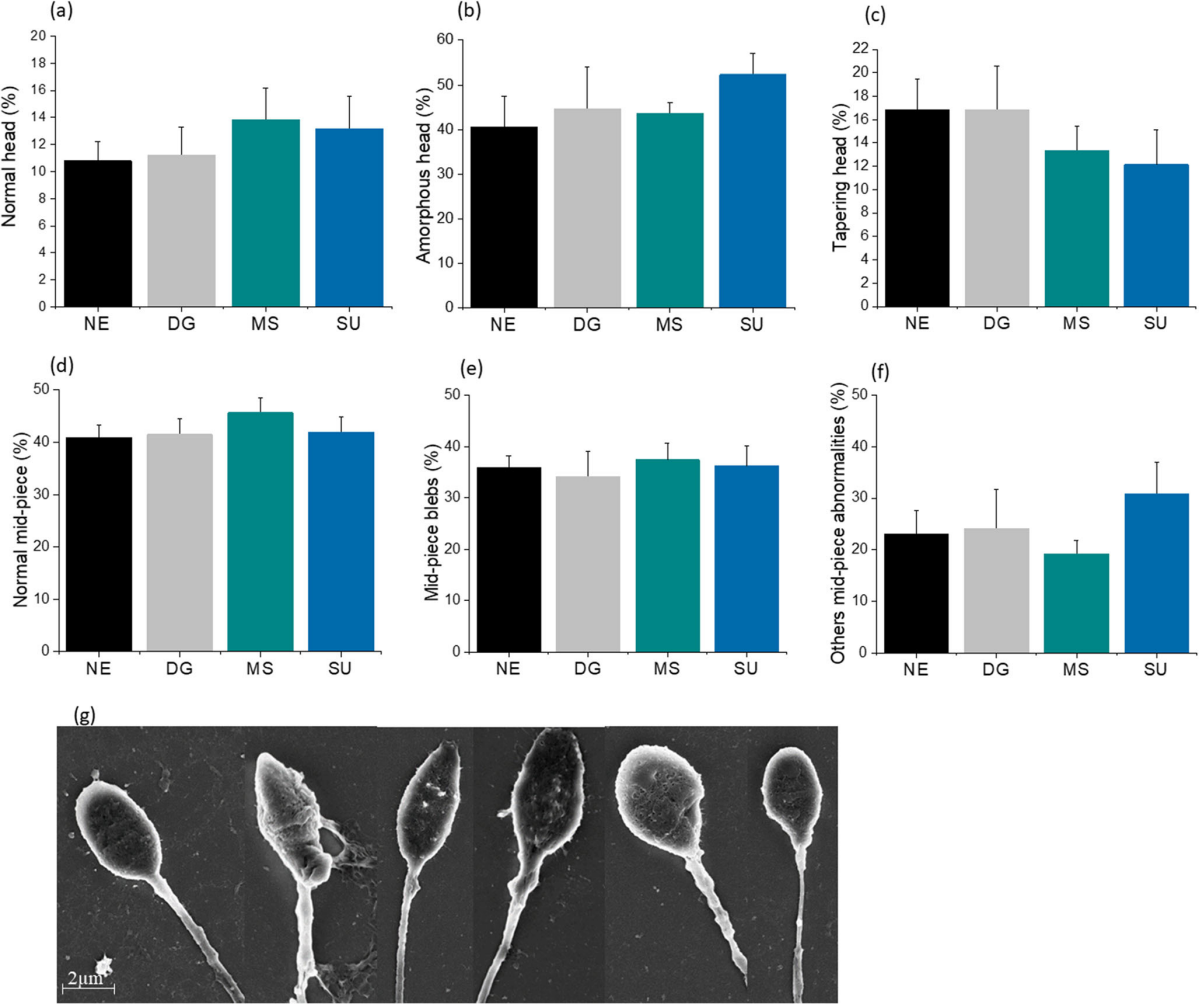
Ultrastructural analysis of processed sperm fraction. **a**–**c** Various forms of head abnormalities observed under scanning electron microscope (SEM). **d**–**f** Various mid-piece abnormalities found in the processed fraction. **g** Representative SEM images showing normal and abnormal forms. Please note that the values are not significantly different between the groups

Results from the above experiments so far have suggested that spermatozoa extracted by MS and SU are comparable with respect to total motile sperm, functional ability, and DNA integrity. On the other hand, DG-extracted sperm quality was found inferior to MS and SU fractions. Since MS is a non-centrifugation-based technique, we speculated that morphology of spermatozoa extracted by using this technique is inferior to other two methods as centrifugation alone or in combination with gradients, as SU and DG techniques have the ability to reduce morphologically abnormal spermatozoa. Morphology analysis of extracted spermatozoa by using the Shorr technique did not show significant variation across the three methods, though DG and MS methods have extracted moderately higher number of spermatozoa with normal head morphology, but the differences were not statistically significant (Supplementary figure 5). Furthermore, ultrastructural analysis using scanning electron microscopy also confirmed a moderate increase in the number of spermatozoa with normal head and mid-piece in the MS group (Fig. 5a–e). On the other hand, amorphous forms and other mid-piece abnormalities were marginally higher in SU-extracted spermatozoa (Fig. 5b–f). However, no statistical power was demonstrated between any of these groups.

## Discussion

We demonstrate that the MS-based device used for the ejaculate processing allows the selection of clinically usable, functionally competent spermatozoa with a significant reduction in the levels of DNA fragmentation. All functional parameters tested using split fractions of the ejaculates have shown that the sperm fraction can be enriched similar to the SU technique. On the other hand, density gradient centrifugation resulted in reduced motility, mitochondrial damage, and less number of capacitated spermatozoa in the processed fraction compared with MS and SU. Furthermore, the ability of DG to eliminate the number of spermatozoa carrying IDL was significantly lower than those of MS and SU methods.

MS-based sperm processing offers an alternative to traditional sperm selection techniques wherein enriched fraction of spermatozoa is isolated from an unprocessed ejaculate [15, 16]. The MS device MIGLIS®, a commercial product from Menicon Life Science, Japan, was used in this study. The readily available disposable device is relatively inexpensive and intended to use without the need for special technical skills or equipment, hence best suited for sperm preparation for IUI. Apart from the improvement in motility and DNA integrity via processing by using a MS device, the TMS was also comparable to other two techniques, reflecting the selective nature of the device without compromising the recovery rate. In our study, only split samples were processed to understand the relative benefit of the techniques employed. Since the TMS is > 5 million and the recovery rate is approximately 50 in MS, it has potential to extract adequate number of spermatozoa to establish pregnancy in IUI programmes. Nonetheless, a single MS device is capable of processing an ejaculate up to 3 mL, which is higher than those of the published centrifugation-free devices [11, 12], and therefore, will maximize the sperm recovery, which can be used for IUI.

Protein tyrosine phosphorylation is a key intracellular signalling event regulating sperm function and is an indicator of capacitation and essential for sperm to correctly undergo acrosome reaction. [25–27] Seminal fluid–derived proteins attached to the sperm surface can inhibit the process of capacitation by negatively affecting the hyperactive motility and protein tyrosine phosphorylation [28]. The seminal plasma was in contact with spermatozoa throughout the MS selection, and our results have shown that the protein tyrosine phosphorylation level was affected in the processed fraction; however, the level was significantly higher than that of the DG group. Furthermore, sperm motility at 6 and 18 h post-selection was significantly higher in MS when compared with that of DG, suggesting that exposure of seminal plasma and seminal proteins to spermatozoa during MS selection has not compromised the functionality.

Semen processing using the gradient centrifugation and swim-up procedure in this study took 40 and 80 min respectively, whereas the MS device needed 65 min to complete the process in a single step without any centrifugation. Reducing the treatment time and eliminating the centrifugation step may prevent iatrogenic damage to sperm functional and genetic integrity [4]. Though few studies have suggested that centrifugation of semen samples is associated with the generation of increased reactive oxygen species and a high DNA damage [8], the others failed to show such association [18, 29]. These conflicting observations may be related to the extent of DNA fragmentation in sperm cells, duration of the ejaculatory abstinence, quality of the ejaculate, and/or the technique used for sperm selection [30–32]. The swim-up method significantly reduces sperm DNA fragmentation rates and may have prognostic value on IUI in patients with decreased sperm DNA integrity [8, 33], which is in agreement with our observation. However, SU is relatively time-consuming, is associated with sperm seminal plasma exposure during centrifugation, and needs several rounds of centrifugation steps. On the other hand, semen processing by DG centrifugation did not improve sperm apoptotic DNA fragmentation rate [34]. Importantly, a recent study has evaluated the efficacy of SU and DG in removing DNA-damaged spermatozoa and found increased sperm DNA damage during DG and SU in few patients [35]. Our results also did not show any benefits of DG in reducing the DNA-fragmented sperm from both spontaneous and IDL-carrying ejaculates. Though, several techniques exist to test sperm DNA fragmentation such as the sperm chromatin structure assay (SCSA), the sperm chromatin dispersion (SCD) test, the TUNEL assay, and the single-cell gel electrophoresis (Comet) assay, the TUNEL assay and the Comet assay have shown better predictive value [36]. Since the TUNEL test is technically simple with less statistical robustness [37], we have employed the TUNEL assay in this study. DNA damage might be a result of mitochondrial impairment following an apoptotic cascade, [38] and motile spermatozoa can reach the bottom of the conical tube even in the presence of mitochondrial impairment and DNA damage during DG [39]. This observation is in agreement with our results, where both mitochondrial and DNA damages were higher in DG- than in MS-extracted spermatozoa. At this juncture, we would like to state that mitochondrial membrane potential measured using rhodamine 123 is not a sensitive marker, hence it is worth looking into the mitochondrial respiration by studying mitochondria complexes [40].

Based on the results observed in our study, we argue that the MS-based device used here is significantly more effective than the DG device in regard to enrichment of functional and genetic integrity in the processed fraction. Though the functional ability and genetic integrity of MS- and SU-selected spermatozoa were comparable, due to involvement of multiple centrifugation steps, SU-processed spermatozoa are at increased risk of experiencing iatrogenic damage [41]. Thus, the MS method which involves just two pipetting steps is considerably more efficient than the DG and SU techniques.

Ultrastructural analysis of spermatozoa provides a valuable tool to address subtle changes in sperm morphology which cannot be identified by using conventional staining techniques. It has been shown that the artefacts of sperm ultrastructural morphology may be associated with sperm structural fragility and preparation conditions. Spermatozoa from fertile males with centrifugation of 600*g* for washing sperm exhibited more damage to the mid-piece than those with the 300*g* [42]. The centrifugation speed employed in the present study (500×*g* and 300×*g*) is moderately higher than the WHO [17] recommendations of 300–400×*g*/15–30 min for the initial wash and 200×*g*/4–10 min for subsequent washes in DG. Similarly, the SU technique involved two rounds of centrifugation washing instead of single-step washing and overlying followed by others. Hence, it is possible that one of the reasons for seeing increased ultrastructural changes in the mid-piece of the SU fraction could be attributed to the centrifugation steps. However, we could not establish any statistical differences in these observations possibly due to limited sample size.

Though several other non-centrifugation techniques based on microfluidic and chemotaxis principles are used for the selection of clinically usable, highly motile sperm with nearly undetectable levels of DNA fragmentation [11–13], only intracytoplasmic sperm injection (ICSI) is the possible way of fertilization due to relatively small number of spermatozoa collected at the end. IUI is useful only when total motile sperm count (TMSC) is > 5 million [43], and our results have shown that MS can easily extract a minimum of 5 million TMSC from the normozoospermic split fractions. However, at this juncture, it is to be noted here with great emphasis that a major limitation of this study is that only normozoospermic ejaculates were used, as a consequence of which, the study cannot reveal the ability of the MS device in extracting adequate number of enriched spermatozoa from the poor-quality ejaculates. In this study, the EBSS + sodium bicarbonate buffering system was used in all three techniques to make the observations comparable. However, use of CO_2_-free incubation is the most practical approach in a physician’s office or in a limited-resource setting which can be accomplished by optimizing the medium buffering system (MOPS or HEPES) and a simple dry bath incubation.

In conclusion, the MS-based device offers a centrifugation-free, efficient, and reliable sperm selection method, hence suitable for partially equipped intra-uterine insemination (IUI) laboratories or office IUI programmes. Further research should focus on the safety and clinical usefulness of the device in medically assisted conception programmes in general and IUI in specific.

## Electronic Supplementary Material

ESM 1 (PNG 464 kb).

High Resolution image (TIF 233 kb).

ESM 2 (PNG 705 kb).

High Resolution image (TIF 341 kb).

ESM 3 (PNG 353 kb).

High Resolution image (TIF 187 kb).

ESM 4 (PNG 743 kb).

High Resolution image (TIF 391 kb).

ESM 5 (PNG 170 kb).

High Resolution image (TIF 86 kb).

## Data Availability

The data and material that support the findings of this study are available from the corresponding author upon request.
