# Supplementary figures and images for: A Simple, Centrifugation-Free, Sperm-Sorting Device Eliminates the Risks of Centrifugation in the Swim-Up Method While Maintaining Functional Competence and DNA Integrity of Selected Spermatozoa
Source: Reprod Sci. 2020 Jul 30;28(1):134–43. doi: 10.1007/s43032-020-00269-5 (PMC7782414; doi:10.1007/s43032-020-00269-5)

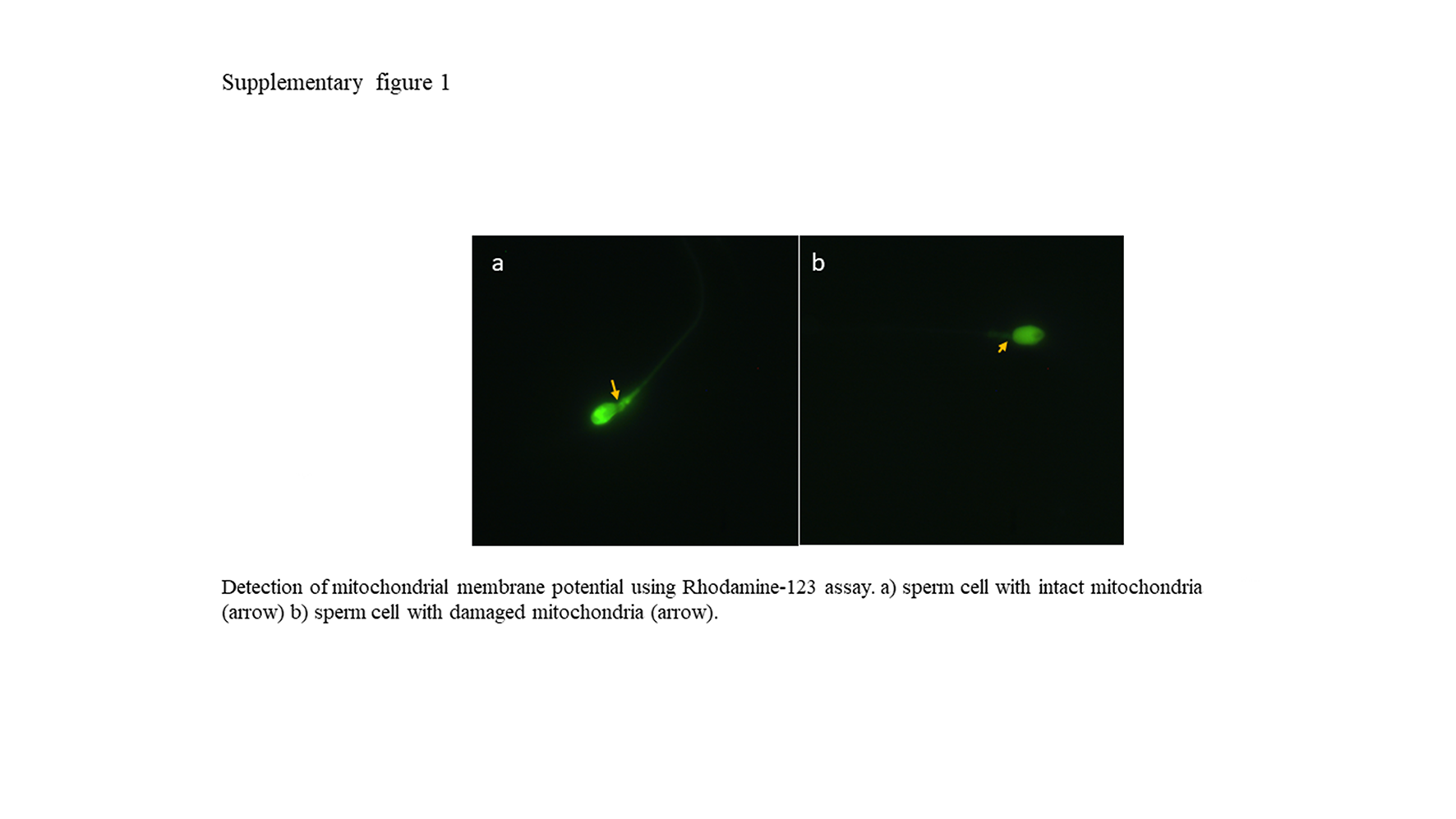

Supplement: Supplementary file 1 — (PNG 464 kb). [file 43032_2020_269_Fig6_ESM.png]

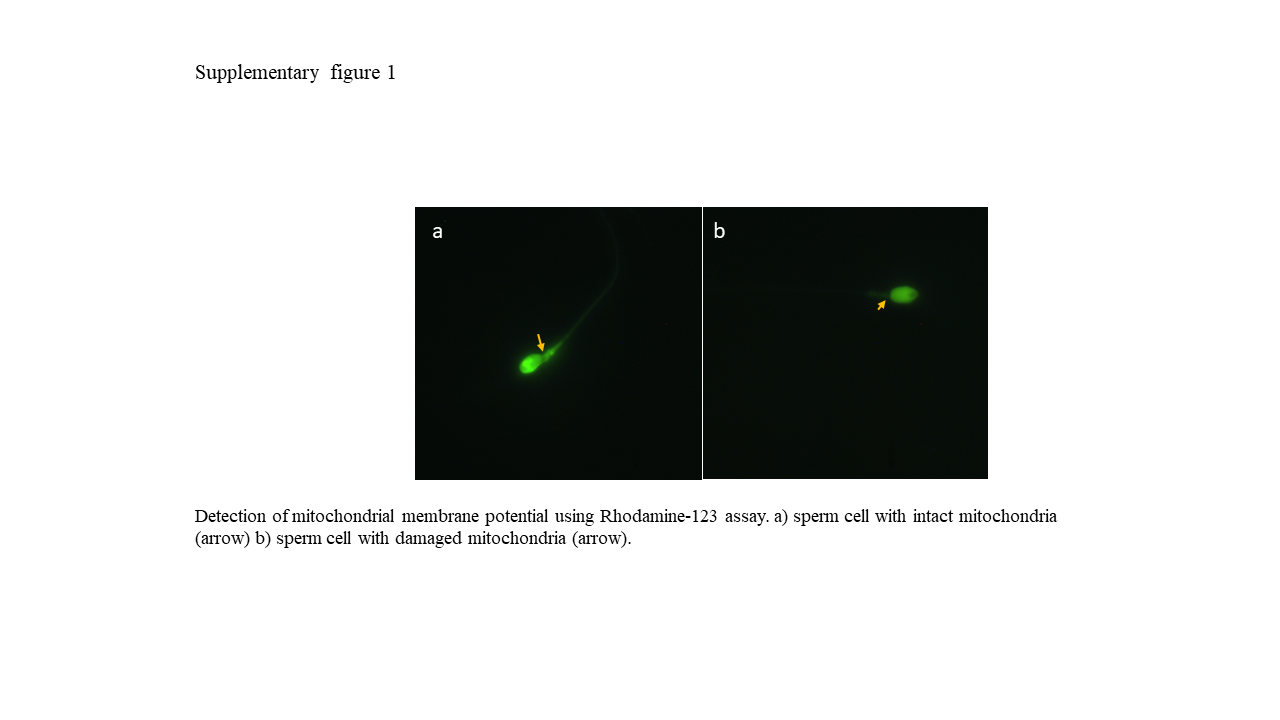

Supplement: Supplementary file 2 — High Resolution image (TIF 233 kb). [file 43032_2020_269_MOESM1_ESM.tif]

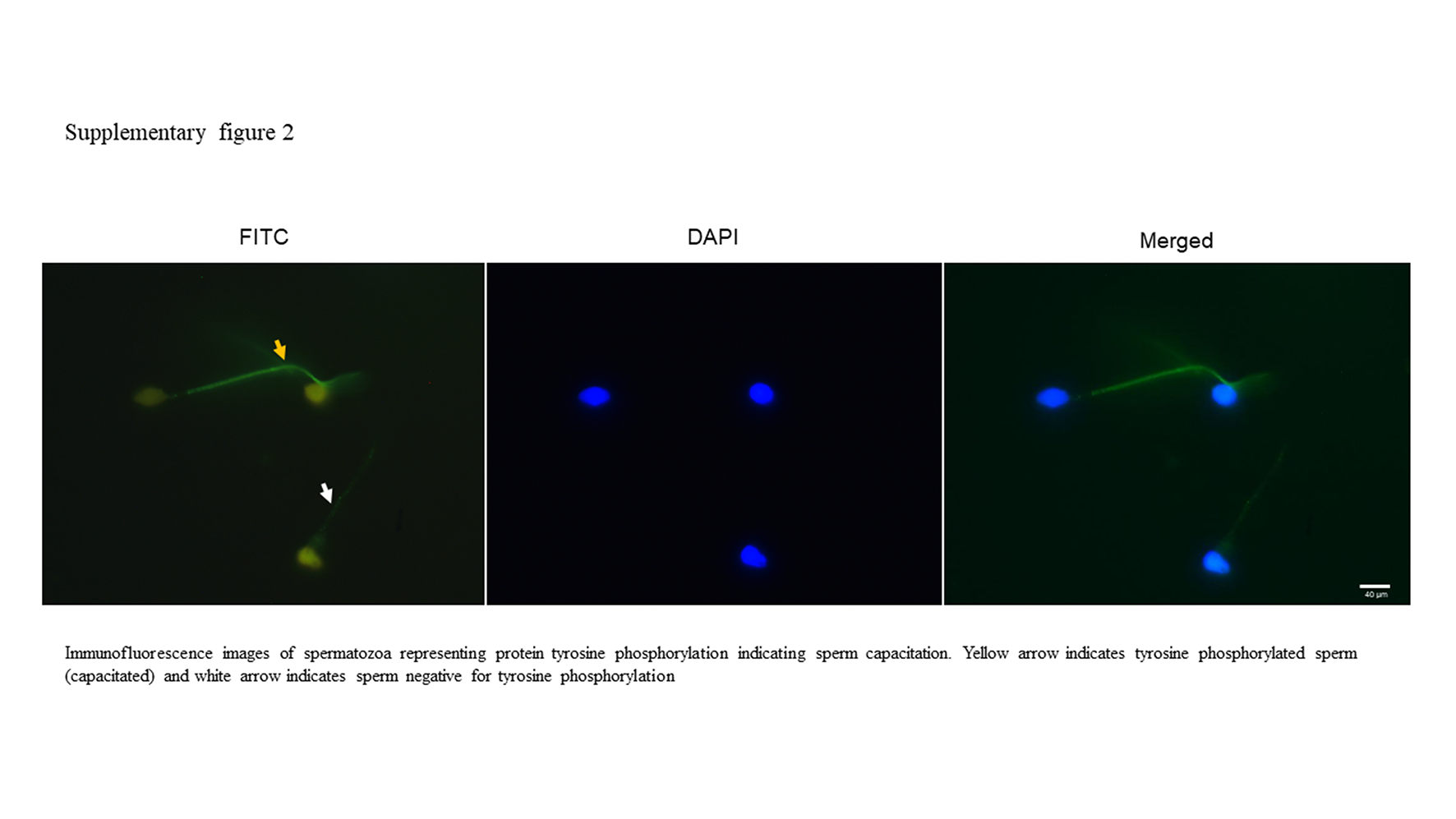

Supplement: Supplementary file 3 — (PNG 705 kb). [file 43032_2020_269_Fig7_ESM.png]

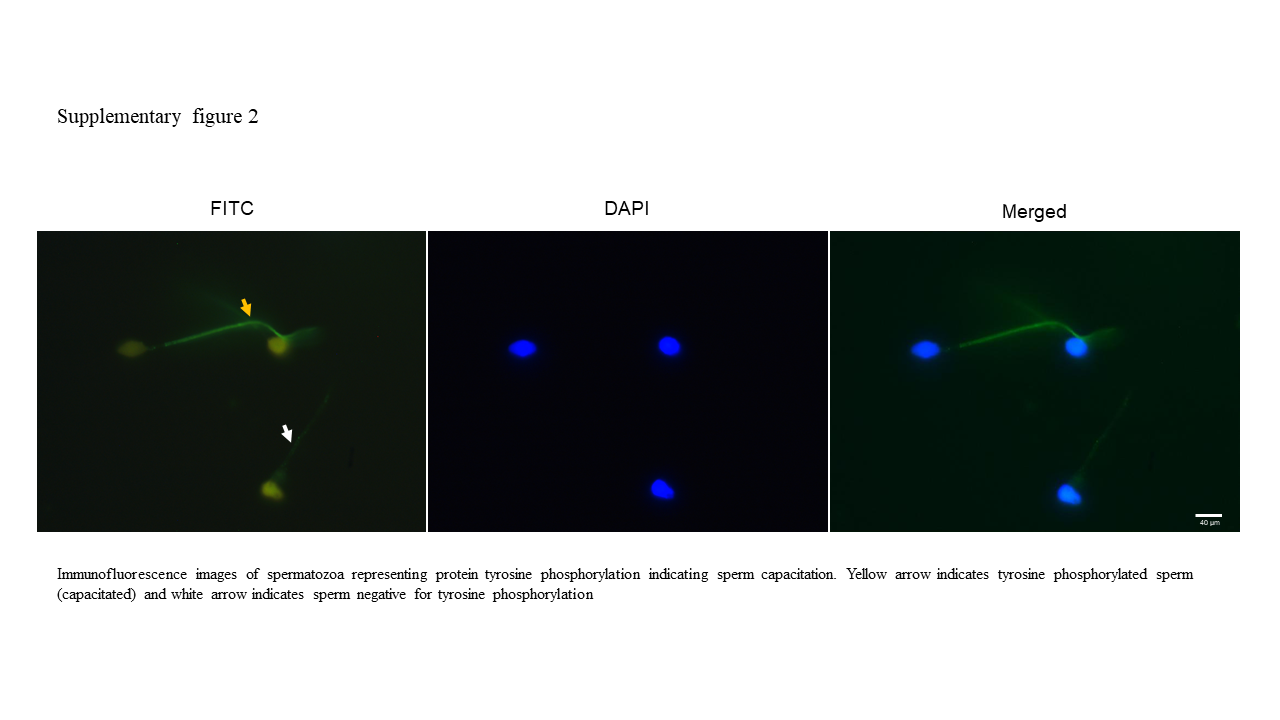

Supplement: Supplementary file 4 — High Resolution image (TIF 341 kb). [file 43032_2020_269_MOESM2_ESM.tif]

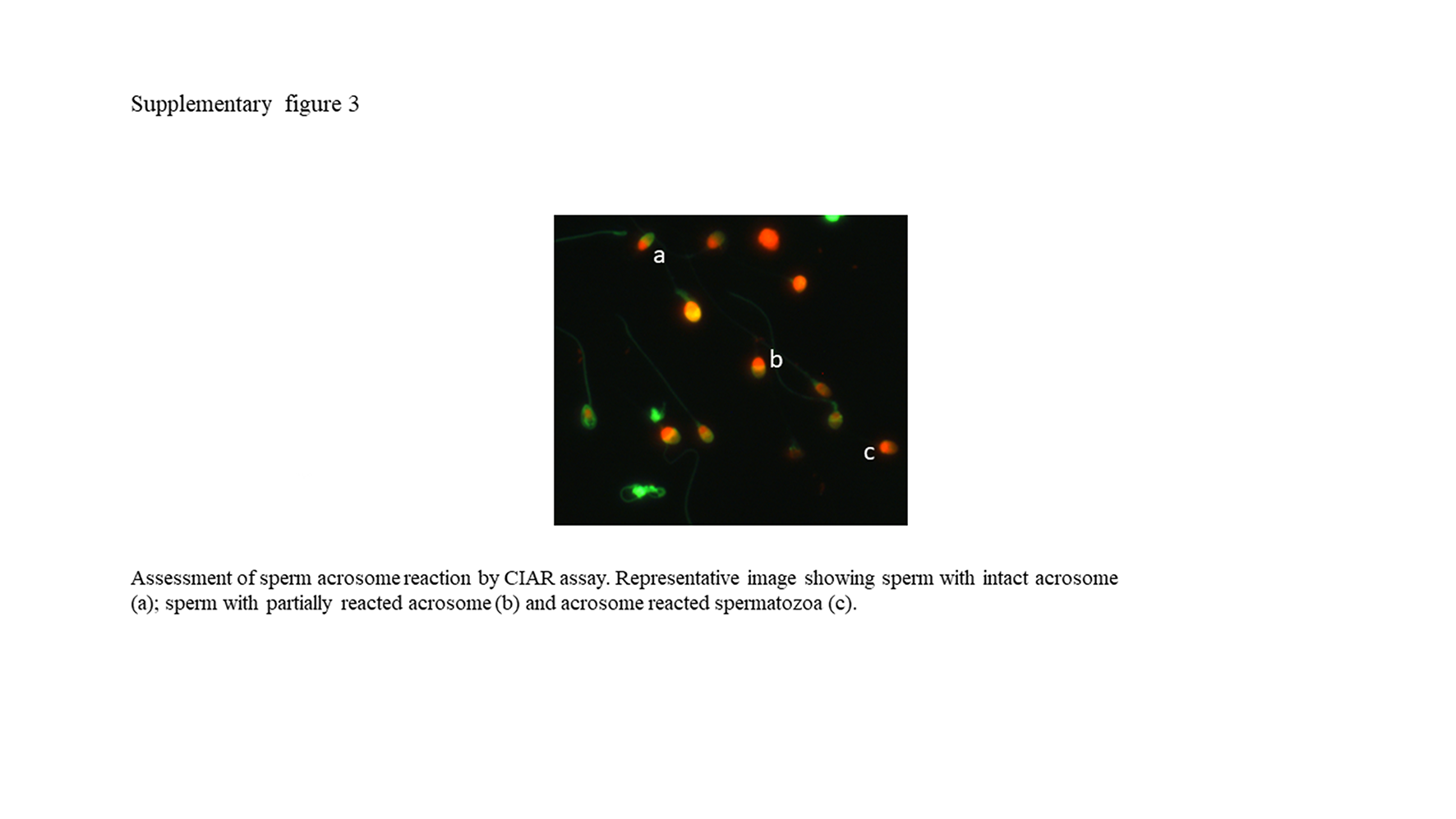

Supplement: Supplementary file 5 — (PNG 353 kb). [file 43032_2020_269_Fig8_ESM.png]

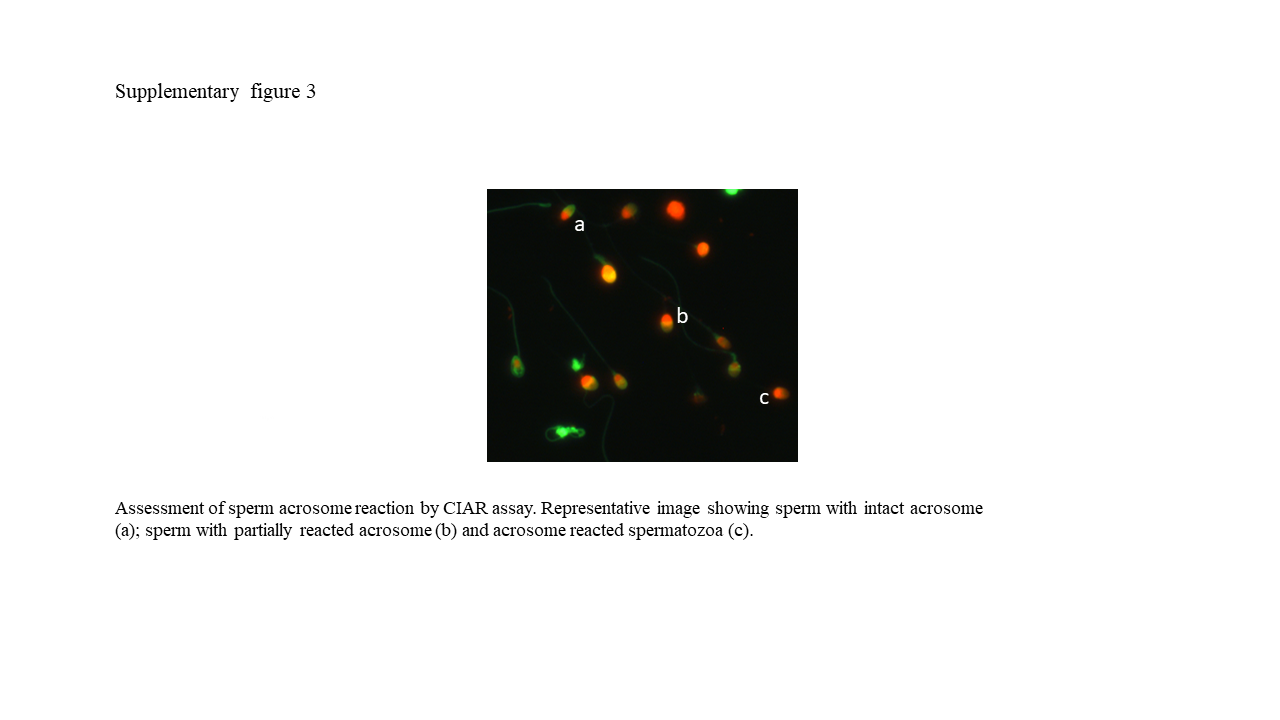

Supplement: Supplementary file 6 — High Resolution image (TIF 187 kb). [file 43032_2020_269_MOESM3_ESM.tif]

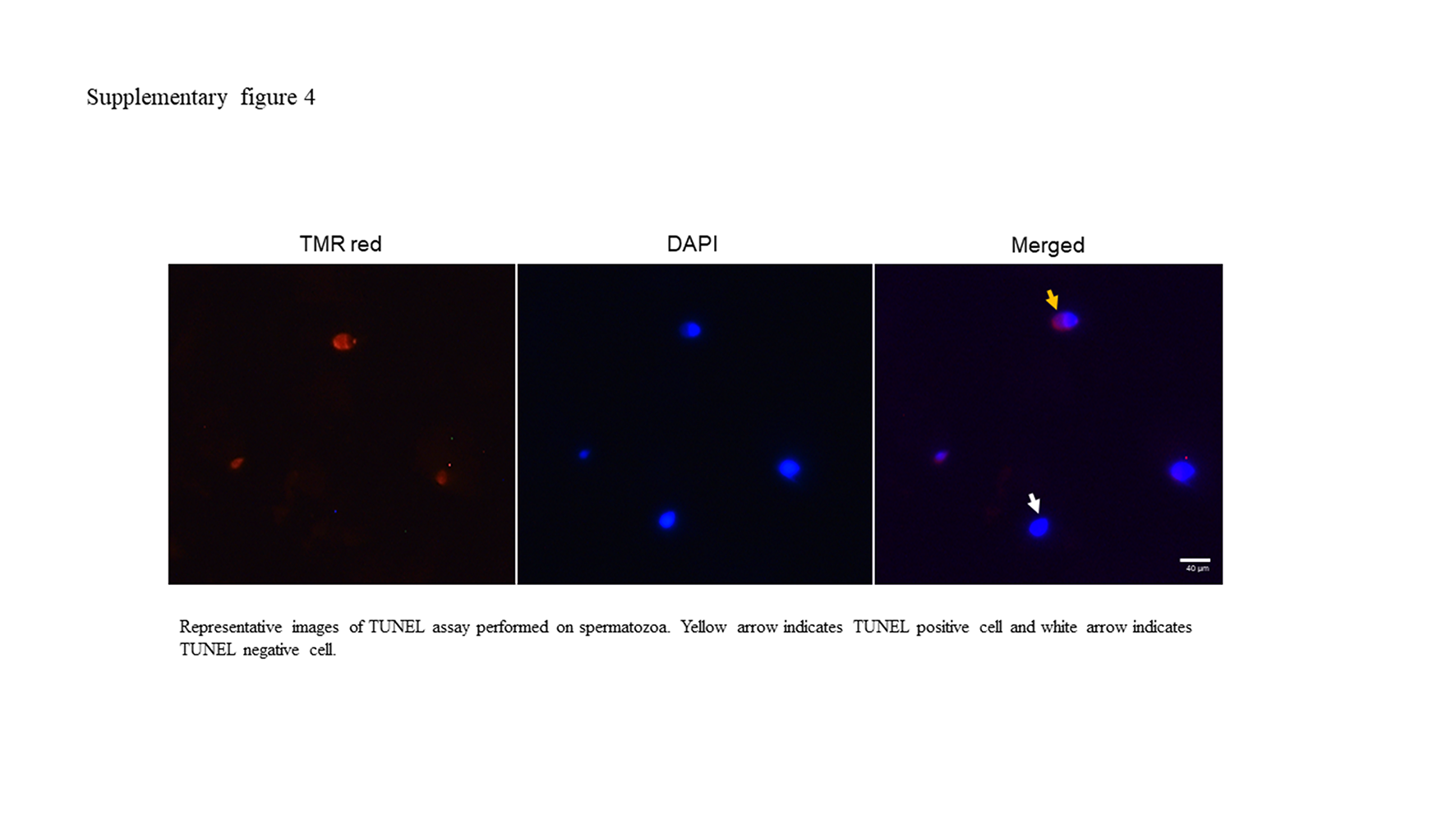

Supplement: Supplementary file 7 — (PNG 743 kb). [file 43032_2020_269_Fig9_ESM.png]

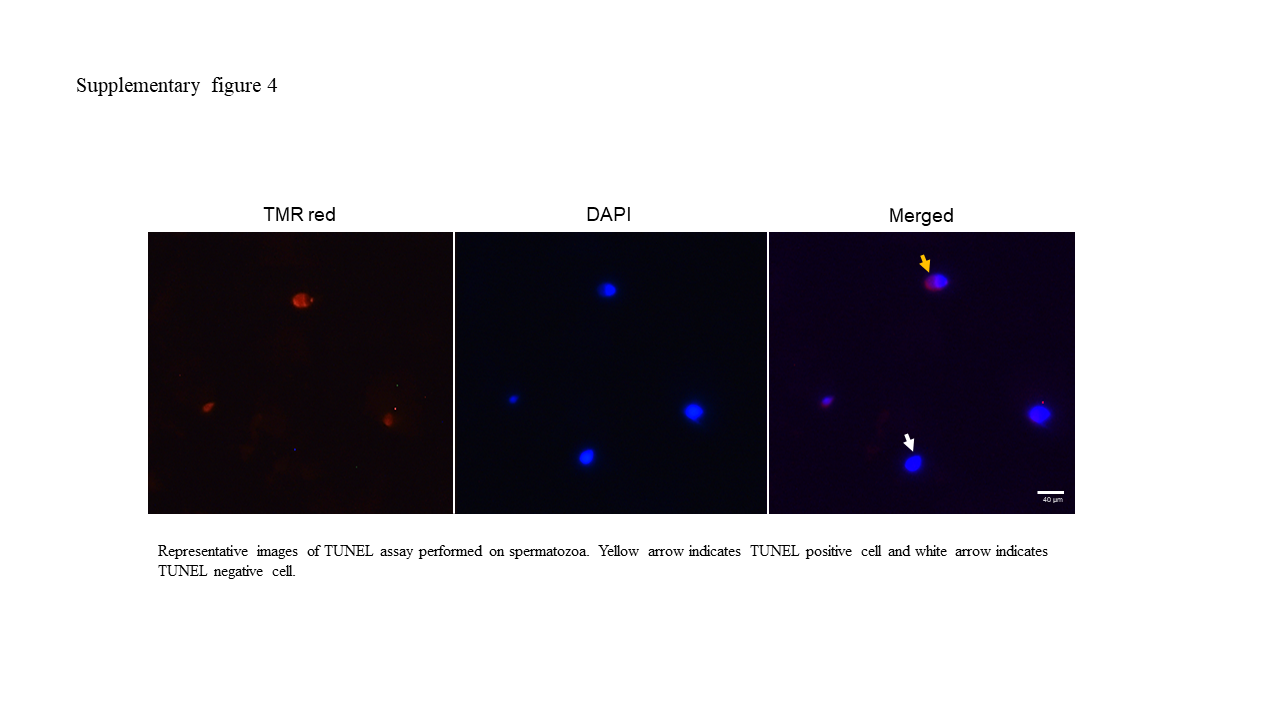

Supplement: Supplementary file 8 — High Resolution image (TIF 391 kb). [file 43032_2020_269_MOESM4_ESM.tif]

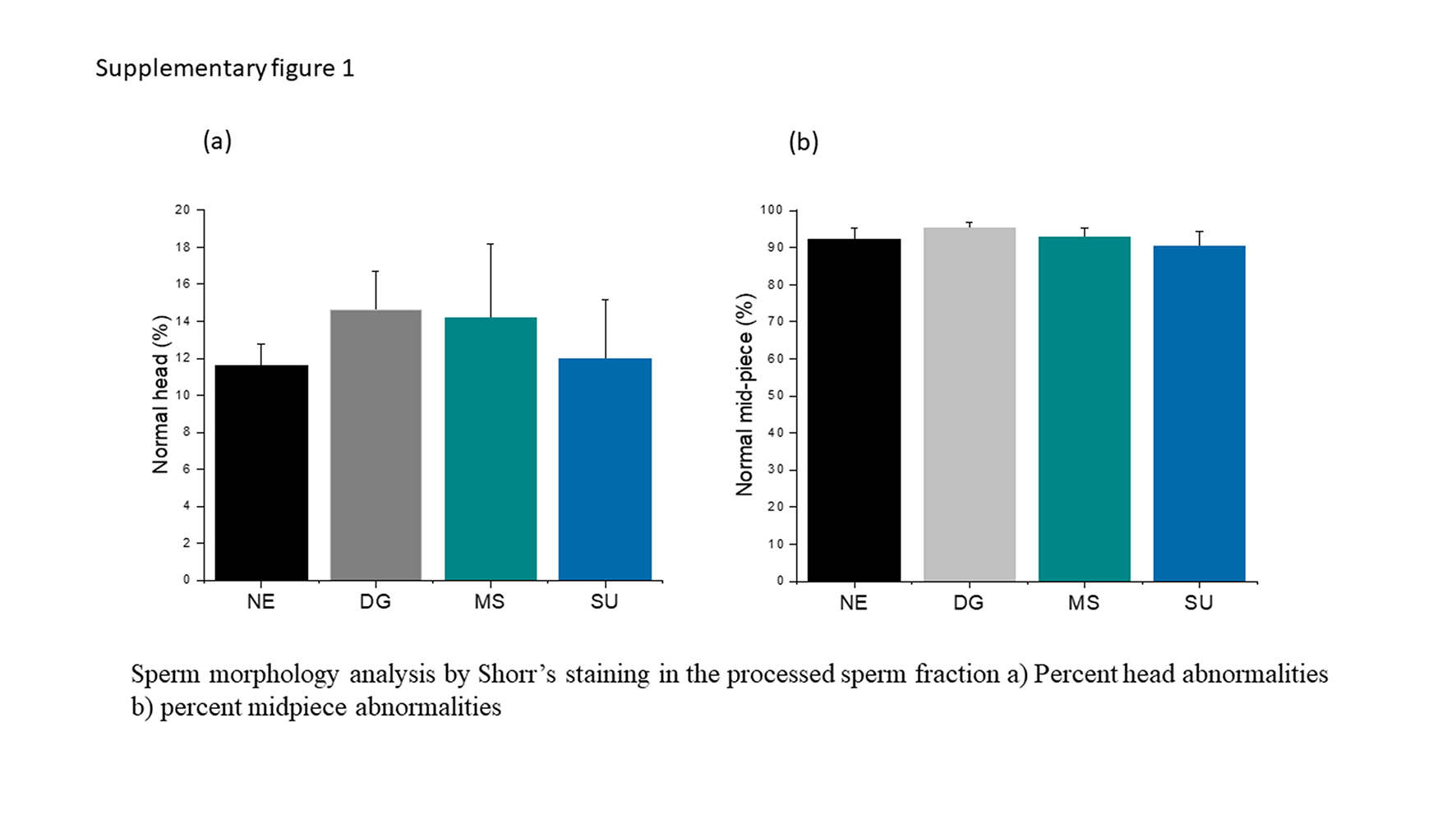

Supplement: Supplementary file 9 — (PNG 170 kb). [file 43032_2020_269_Fig10_ESM.png]

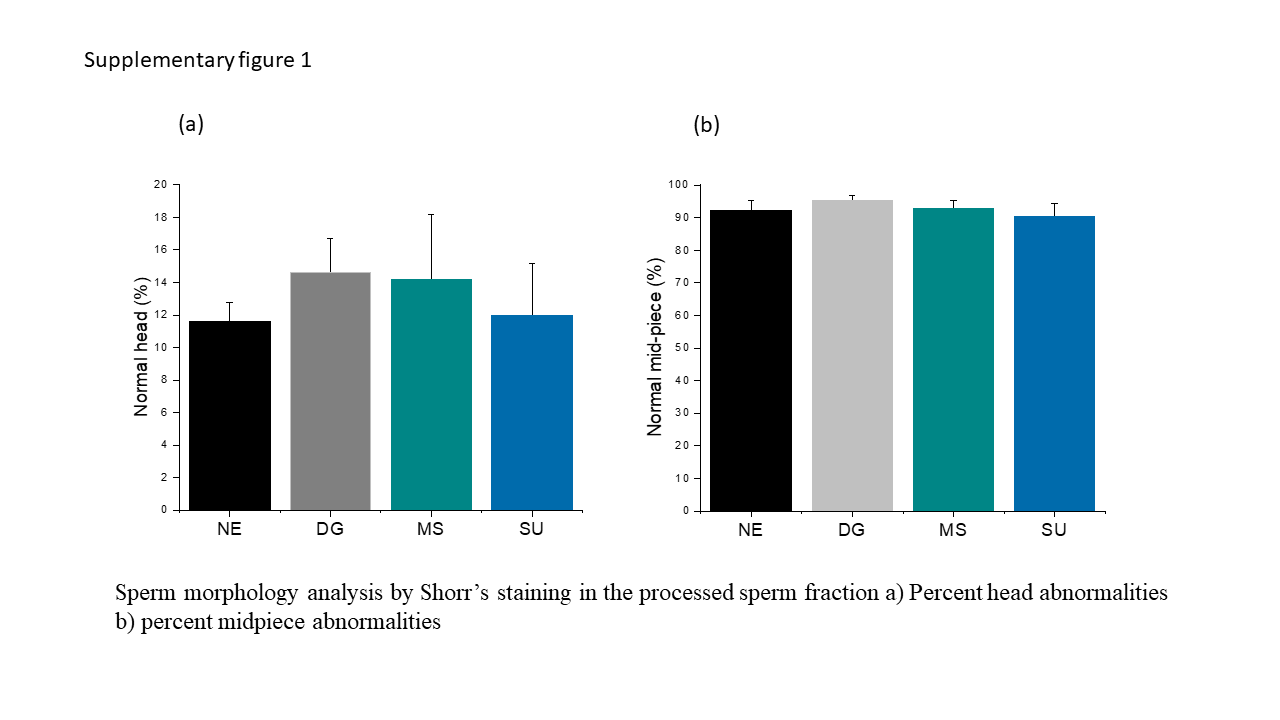

Supplement: Supplementary file 10 — High Resolution image (TIF 86 kb). [file 43032_2020_269_MOESM5_ESM.tif]
